# LDL Promotes Disorders in β-Cell Cholesterol Metabolism, Implications on Insulin Cellular Communication Mediated by EVs

**DOI:** 10.3390/metabo12080754

**Published:** 2022-08-16

**Authors:** Lizbeth Guevara-Olaya, Brenda Chimal-Vega, César Yahel Castañeda-Sánchez, Leslie Y. López-Cossio, Angel Pulido-Capiz, Octavio Galindo-Hernández, Raúl Díaz-Molina, Josefina Ruiz Esparza-Cisneros, Victor García-González

**Affiliations:** 1 Departamento de Bioquímica, Facultad de Medicina Mexicali, Universidad Autónoma de Baja California, Mexicali 21000, BC, Mexico; 2 Laboratorio Multidisciplinario de Estudios Metabólicos y Cáncer, Facultad de Medicina Mexicali, Universidad Autónoma de BC, Mexicali 21000, BC, Mexico; 3 Laboratorio de Biología Molecular, Facultad de Medicina Mexicali, Universidad Autónoma de Baja California, Mexicali 21000, BC, Mexico; 4 Departamento de Nutrición, Facultad de Medicina, Universidad Autónoma de Baja California, Mexicali 21000, BC, Mexico

**Keywords:** β-cells, LDL, insulin, cholesterol, metainflammation, PDX-1, auraptene, extracellular vesicles

## Abstract

Dyslipidemia is described as a hallmark of metabolic syndrome, promoting a stage of metabolic inflammation (metainflammation) that could lead to misbalances in energetic metabolism, contributing to insulin resistance, and modifying intracellular cholesterol pathways and the renin–angiotensin system (RAS) in pancreatic islets. Low-density lipoprotein (LDL) hypercholesterolemia could disrupt the tissue communication between Langerhans β-cells and hepatocytes, wherein extracellular vesicles (EVs) are secreted by β-cells, and exposition to LDL can impair these phenomena. β-cells activate compensatory mechanisms to maintain insulin and metabolic homeostasis; therefore, the work aimed to characterize the impact of LDL on β-cell cholesterol metabolism and the implication on insulin secretion, connected with the regulation of cellular communication mediated by EVs on hepatocytes. Our results suggest that β-cells can endocytose LDL, promoting an increase in *de novo* cholesterol synthesis targets. Notably, LDL treatment increased mRNA levels and insulin secretion; this hyperinsulinism condition was associated with the transcription factor PDX-1. However, a compensatory response that maintains basal levels of intracellular calcium was described, mediated by the overexpression of calcium targets PMCA1/4, SERCA2, and NCX1, together with the upregulation of the unfolded protein response (UPR) through the activation of IRE1 and PERK arms to maintain protein homeostasis. The LDL treatment induced metainflammation by IL-6, NF-κB, and COX-2 overexpression. Furthermore, LDL endocytosis triggered an imbalance of the RAS components. LDL treatment increased the intracellular levels of cholesterol on lipid droplets; the adaptive β-cell response was portrayed by the overexpression of cholesterol transporters ABCA1 and ABCG1. Therefore, lipotoxicity and hyperinsulinism induced by LDL were regulated by the natural compound auraptene, a geranyloxyn coumarin modulator of cholesterol-esterification by ACAT1 enzyme inhibition. EVs isolated from β-cells impaired insulin signaling via mTOR/p70S6Kα in hepatocytes, a phenomenon regulated by auraptene. Our results show that LDL overload plays a novel role in hyperinsulinism, mechanisms associated with a dysregulation of intracellular cholesterol, lipotoxicity, and the adaptive UPR, which may be regulated by coumarin-auraptene; these conditions explain the affectations that occur during the initial stages of insulin resistance.

## 1. Introduction

The relationship among obesity, high levels of Low-Density Lipoproteins (LDL), low levels of High-Density Lipoprotein (HDL), insulin resistance, and arterial hypertension involve a high lipid diet and adipose tissue mass gain, leading to the development of low-grade systemic inflammation. These conditions are associated with the synthesis and release of pro-inflammatory cytokines TNF-α, IL-6, and IL-1 [1], a phenomenon-denominated metainflammation, wherein an increase in serum LDL occurs. Moreover, excessive lipid accumulation could contribute to dysfunctional insulin secretion in Langerhans β-cells [2,3].

During the early stage of type 2 diabetes mellitus (T2DM), the synthesis and secretion of insulin by islet β-cells are enhanced, coupled with endoplasmic reticulum (ER) stress [4], and alterations in the management of lipid metabolism. Elevated levels of plasmatic insulin may contribute to nonalcoholic fatty liver disease (NAFLD) [5]. The presence of NAFLD promotes the transition from metabolically healthy to metabolically unhealthy obese patients [6], possibly impacting insulin signaling and lipid metabolism. LDL-hypercholesterolemia could promote a deleterious response to a systemic metabolism, considering the presence of the metainflammation [1,7]. However, the effect of these alterations on β-cell physiology is not yet elucidated. In vitro assays suggest hypercholesterolemia could promote islet dysfunction [2], modifying insulin secretion. Under this condition, adaptive responses such as ER stress and calcium homeostasis would be triggered.

ER stress triggers the unfolded protein response (UPR), and this pathway consists of three major signaling branches initiated by ATF6, PERK, and IRE1, which function as sensors of misfolded protein overload in the ER lumen, activating transcription factors ATF6α, CHOP, and XBP1s, respectively [8]. UPR promotes the folding capacity by increasing the production of chaperones and enzymes of lipid synthesis [9]; likewise, ER luminal Ca^2+^ regulates protein synthesis and folding [10]. Moreover, cellular perturbations induced by lipotoxicity can also lead to ER stress [11]. Altogether, these mechanisms maintain cell homeostasis [12]. However, the role of UPR in LDL-hypercholesterolemia and the impact on intracellular cholesterol and insulin metabolism in β-cells has not been elucidated.

Cells obtain cholesterol exogenously from lipoproteins and de novo biosynthesis [13]. In this sense, the ER serves as the primary site of cholesterol biosynthesis. The ER-resident 3-hydroxy-3-methyl-glutaryl-coenzyme A reductase (HMG-CoA reductase) controls the rate-limiting reaction, for which the levels are modulated by the master regulator sterol regulatory element-binding protein 2 (SREBP2). Critically, the cellular cholesterol concentrations are highly regulated; ATP-binding cassette (ABC) transporters ABCA1 and ABCG1 are the central modulators of cholesterol homeostasis through cholesterol exportation [14]. Indeed, ABCA1 downregulation induced cholesterol accumulation and progressive lipotoxicity [15,16]. Then, a close association between cholesterol metabolism, calcium dysregulation, lipotoxicity, and ER stress could be present.

In results obtained by our group, treatment with saturated fatty acids triggered UPR. It promoted lipotoxicity through the dysregulation of calcium modulators such as plasma membrane Ca^2+^-ATPase (PMCA1/4), the Na^+^/Ca^2+^ exchanger (NCX1), and Sarco/endoplasmic reticulum Ca^2+^-ATPase (SERCA2) in β-cells [17]. Therefore, cells maintain subtle mechanisms to regulate cytoplasmic calcium concentrations, considering its critical role in insulin exocytosis; moreover, imbalances in calcium levels are triggering conditions of UPR [18]. Indeed, a connection between the calcium-binding protein involved in maintaining endoplasmic reticulum (ER) Ca^2+^ has been established with the UPR under high-fat diet-induced lipotoxicity in β-cells [19].

Related to high levels of LDL and insulin processing, local mechanisms can be affected, for instance, the ones that depend on the signaling of the renin–angiotensin system (RAS). RAS hyperactivity has been recognized as a metabolic regulator in Langerhans islets [20]. The angiotensin II receptor type 1 (AT1R) overexpression is associated with endothelial dysfunction, oxidative stress, and angiogenesis [21]. A parallel axis is performed by carboxypeptidase ACE2, which hydrolyzes angiotensin II, providing a compensatory mechanism to counteract the detrimental effects of hyperactive RAS [20]. ACE2 has been described to improve glycemia in angiotensin II-infused mice [20], and hepatocyte nuclear factor 1-alpha (HNF1α) induces the expression of ACE2 in the pancreas [22], which could improve glucose homeostasis. Then, a regulatory mechanism could exist between HNF1α and LDL-hypercholesterolemia connected with metainflammation in β-cells.

Moreover, metainflammation could disrupt processes implicated in tissue communication, especially those involving extracellular vesicles (EVs). EVs are a heterogeneous group of vesicles surrounded by a lipid bilayer secreted by multiple cells, including exosomes, microvesicles, and apoptotic bodies [23]. Modifications in the tissue microenvironment, such as the adipose, pancreas, liver, and muscle, changes in the metabolism status, and exposure to cytokines stimulate the secretion and modification in cargo molecules of EVs, which are associated with pathologic conditions such as insulin resistance, high blood pressure, and metabolic syndrome [24,25]. For instance, EVs secreted by hepatocytes have been shown to induce β-cell proliferation [26]; even more, multi-organ crosstalk with the endocrine pancreas could be mediated by EVs [27]. Primarily, β-cell-dependent mechanisms with hepatocytes could regulate systemic metabolism.

Targeting lipid metabolism pathways that regulate β-cell homeostasis by using small molecules obtained from natural sources may offer potential treatment options for T2DM. For instance, Auraptene (Aur), a geranyloxyn coumarin, has been described as an inhibitor of cholesterol esterification [28], possibly alleviating the harmful effect of cholesterol accumulation in lipid droplets. Therefore, the aim of this work was to characterize the impact of LDL treatment on β-cell cholesterol metabolism and its implication on insulin secretion, connected with the regulation of EVs’ cellular communication between Langerhans β-cells and hepatocytes. Finally, we lead a strategy for their regulation mediated by the coumarin, Auraptene.

## 2. Materials and Methods

### 2.1. Reagents

Cell culture reagents were purchased from Thermo-Fisher Scientific (Waltham, MA, USA), tissue culture plates and plastic materials were obtained from Corning (Corning, NY, USA). RPMI-1640 and DMEM media, trypsin, fetal bovine serum (FBS), penicillin, streptomycin, and amphotericin B were obtained from Thermo-Fisher Scientific. MTT, auraptene (Aur > 98% of purity), and tunicamycin (Tum) were obtained from Merck. Anti-SREBP2, anti-HMGCR, anti-LDL-R, anti-β-adaptin, anti-ACE2, anti-HNF1α, anti-AT1R, anti-TMPRSS2, anti-AT1R, anti-PDX-1, anti-Bcl-2, anti-BAX, anti-Lamin B, anti-GAPDH, anti-PMCA1/4, anti-SERCA2, anti-NCX1, anti-CHOP, anti- NF-κB, anti-IL-6, anti-COX-2, anti-XBP1s, anti-PDI, anti-ABCA1, anti-ABCG, anti-PPARα, anti-ACAT, and anti-β-actin antibodies were obtained from Sta. Cruz Biotechnology (Dallas, TX, USA). Anti-XBP1s was purchased from Abcam (Cambridge, UK).

### 2.2. Cell Cultures

The established β-cell line RIN-m5F (American Type Culture Collection, ATCC, Manassas, VA, USA) was grown in RPMI-1640 medium supplemented with 10% FBS, 10 U/mL penicillin, 10 µg/mL streptomycin, and 25 µg/mL amphotericin B. The culture medium was changed every 3 to 4 days and also passaged once per week, according to ATCC recommendations. In addition, C9-derived rat liver cells (ATCC) were grown in the DMEM medium using 10% FBS, 10 U/mL penicillin, 10 µg/mL streptomycin, and 25 µg/mL amphotericin B. The culture medium was changed every 1 to 2 days and also passaged twice per week. Cultures were maintained at 37 °C in a humidified atmosphere of 95% air and 5% CO_2_.

### 2.3. LDL Isolation and Fluorescent Labeling

Human plasma samples were obtained from a healthy donor that signed informed consent. The protocol was designed and carried out according to the Declaration of Helsinki and registered in the Research Ethics Committee of Facultad de Medicina Mexicali (FMM/CEI/0010/2020-2). Plasma density was adjusted to 1.019 g/mL with KBr and centrifuged at 345,500× *g* for 160 min using an S140-AT 2555 rotor; the VLDL- and IDL-containing layer was discarded. Subsequently, density was adjusted to 1.053 g/mL and centrifuged at 377,000× *g* for 200 min; the upper fraction-containing LDL was recovered. The HDL fraction was isolated, adjusted to a 1.21 g/mL density, and centrifuged at 377,000× *g* for 180 min. LDL preparation was dialyzed against 150 mM NaCl and filtered through 0.45 μm. LDL protein concentration was measured with the bicinchoninic acid (BCA) assay (Thermo Fisher Scientific). For the experimentation, a defined volume of buffer (NaCl 150 mM, EDTA 0.024 mM) corresponding to a dose of 20 µg/mL was evaluated as a control. The quality of the isolates was evaluated by characterization of apolipoproteins apoB-100 and apoA1 (Supplementary Figure S1).

Labeling of LDL fraction was carried out with the dilC18 probe (D3911), which is incorporated in the phospholipid monolayer, through incubation of 10 µL of the probe (2 mg/mL) for each 1 mg of protein-LDL for 18 h at 37 °C, obtaining dil-LDL. Solution density was adjusted to a 1.053 g/mL density and centrifuged at 377,000× *g* for 180 min to recover fluorescent lipoproteins. The fraction was recovered and dialyzed against PBS. In the experiments, the volume of PBS corresponding with a dose of 20 µg/mL was evaluated as a vehicle.

### 2.4. Cell Cytometer Assays

Before internalization experiments, RIN-m5F cultures at 90% confluence were incubated in a FBS-free culture medium. After 1 h of fasting, cells were incubated under increasing dil-LDL concentrations (0–40 µg/mL). After having carried out the experiments in 10-mm wells, cells were washed twice with PBS and recovered in a volume of 200 µL. Cellular characterization was performed in a Beckman–Coulter cytometer Cytoflex; 30,000 events were registered, employing the PC7-A channel to record the dil-LDL fluorescence.

### 2.5. Confocal Microscopy

A LEICA TCS-SP8 confocal scanning biological microscope (LEICA, Germany) was employed in the characterization of the subcellular localization of dil-LDL. RIN-m5F cells were proliferated to 90% confluence and treated with dil-LDL for 20 h. Later, cultured cells were washed with PBS; then the Hoescht probe was added and incubated for 30 min at 37 °C. Finally, cells were washed twice with PBS, fixed with 4% paraformaldehyde for 2 min, and mounted for observation. Macroscopically different zones were recorded, preferentially at the center of the specimens. Images were recorded at excitation/emission wavelengths of 408/430-550 and 552/562-700 nm for detection of Hoescht (blue) and dil-LDL (red), respectively, according to previous work reported by our group [29].

### 2.6. Western Blot (WB) Analysis

Cell cultures were maintained in proliferation until 90% confluence. Afterward, cells were incubated under different treatments. Cells were washed with PBS and lysed with protein lysis buffer (150 mM NaCl, 10 mM Tris, pH 7.4, 1% Triton X-100, 0.5% NP40, EDTA, 0.2 mM sodium orthovanadate, 0.3 μM aprotinin,130 μM bestatin, 14 μM E-64, and 1 μM leupeptin) for 30 min at 4 °C. Subsequently, the lysate was centrifuged at 4100× *g* for 10 min, the supernatant was recovered, and protein was quantified using the BCA assay. Samples (25 μg/lane) from the total protein fraction were analyzed by SDS-polyacrylamide gel electrophoresis (SDS-PAGE) on 10 % gels and subsequently transferred to PVDF membrane (Millipore, MA, USA).

Membranes were blocked with 5% nonfat milk in Tris-buffered saline 0.1%-Tween-20 (TBS-T) for 1 h at 37 °C and incubated at 4 °C overnight with the corresponding primary antibody. Following TBS-T washings, membranes were further incubated for 2 h at 37 °C with the corresponding horseradish peroxidase-conjugated secondary antibodies (HRP). Later, membranes were washed with TBS-T and detected the HRP activity with the Immobilon kit (Millipore, MA, USA).

### 2.7. Cellular Fractionation

Three nuclei analysis and organelle separation protocols were explored: protocol A (sucrose/imidazole), protocol B (mannitol/sucrose), and protocol C (sucrose/HEPES) (Supplementary Figure S7) [29,30]. The most satisfactory method to analyze nuclei was protocol A using sucrose (250 mM) and imidazole (3 mM) pH 7.4 buffer, supplemented with protease and phosphatase inhibitors. Cells were scraped from culture dishes and performed 35 passages through a 30 G syringe. Nuclei separation was verified with Trypan blue staining. For nuclei recovery, lysates were centrifuged at 3400 rpm for 15 min. Then, the two fractions (cytoplasm and nucleus) were lysed with protein lysis buffer for 25 min at 4 °C; both fractions (25 μg/lane) were analyzed by SDS-PAGE and transferred to PVDF membranes.

### 2.8. Isolation of Lipid Droplets and Lipid Quantification

Cells were processed as reported elsewhere [31]. Cells were washed with PBS, scraped from the plate, transferred to a 2 mL tube, and then centrifuged at 2000× *g* for 2 min. The pellet was dissolved in 200 µL of a buffer of 60% sucrose, 10 mM HEPES, 1 mM EDTA, and pH 7.4. After mixing, samples were ice-incubated for 10 min. Next, 800 µL of ice-cold lysis buffer was added and incubated on ice for 10 min. Cells were lysed by 5 passes through a 27-G needle and centrifuged at 100× *g* for 2 min. A mixture of 2 µL of methylene-blue per mL of lysis buffer was prepared; then, 600 µL of this mixture was carefully layered on top of the cell homogenate and centrifuged at 20,000× *g* at 4 °C for 120 min. Tubes were frozen at −70 °C and the dye layer containing lipids was recovered (lipid droplets). Triglyceride and cholesterol content of this fraction were measured (Spin React, S.A.U., Girone, Spain).

### 2.9. Insulin ELISA Assays

Cells were proliferated in 20-mm cell culture plates at a density of 2 × 105 cells/mL, which were processed in a counting-chamber device. Cells were maintained in proliferation for 72 h to reach 90% of confluence, and specific treatments were performed on a volume of 1000 µL. Then, the cell culture medium was recovered and centrifuged for 5 min at 5000 rpm. The supernatant was recovered and diluted in PBS (1/10). Insulin concentrations were quantified with the Ultrasensitive Insulin ELISA kit employing several adaptations according to manufacturer recommendations (80-INSRTU-E01; ALPCO Diagnostics, NH, USA). Absorbance readings were performed at 450 nm and results were reported as ng/mL.

### 2.10. Quantitative PCR for Insulin

Total RNA from RIN-m5F cells was obtained with Trizol reagent, following the supplier’s instructions. cDNA was synthesized using 1 µg of RNA and the Primer Script RT-PCR Kit (Takara Inc, Tokyo, Japan). cDNA concentration was standardized for qPCR with the PowerUp Sybr Green Master Mix 2X (Applied Biosystems, Waltham, MA, USA) according to the manufacturer’s instructions. Primer sequences were insulin forward 5′AGGACCCACAAGTGGAACAACT3′, insulin reverse 5′CAACGCCAAGGTCTTGAAGGT3′, GAPDH forward 5′AGACAGCCGCATCTTCTTGT3′, and GADPH reverse 5′CTTGCCGTGGGTAGAGTCAT3′. qPCR reactions were performed in triplicate using an ABI PRISM 7000 sequence detection system; data were analyzed with the 2^−∆∆Ct^ method with GAPDH as reference calibrator and reported as fold change. In addition, insulin detection was performed in hepatocytes to validate quantification; the detection was negative.

### 2.11. Intracellular Calcium Quantification

Cells were processed as previously described [17], and calcium levels were calculated according to Patel et al. [32]. After treatments, RIN-m5F cells were washed with PBS and incubated at 37 °C with 1.5 µM Fura-2/AM (fluorescent Ca^2+^ indicator) in the opti-MEM medium for 75 min. Subsequently, cells were washed with PBS and incubated for 20 min at 25 °C. Cells were washed with PBS and fluorescence measurements were carried out at 340 and 380 nm excitation wavelengths, and a 510 nm emission wavelength, employing a Cary Eclipse fluorescence spectrophotometer (Santa Clara, CA, USA). *Rmax* was obtained with the addition of Triton X-100 (0.035%) and *Rmin* with the *EGTA *(4.5 mM) incubation [33]. In addition, the insulin-secretagogue effect was evaluated with KCl (30 mM), according to Scullion et al., 2012 [34].

### 2.12. Auraptene Treatment

Auraptene (98% purity) was prepared in a 1 mM DMSO stock solution, then evaluated in a concentration range (1–16 µM) on β-cell cultures and concomitant LDL treatment (20 µg/mL). Based on the results of ABCA1 expression and the impact on cholesterol metabolism, we selected a dose of 4 µM to evaluate the protective effect on several indicators such as insulin secretion, intracellular calcium, and EV secretion. In the experiments, DMSO corresponding with a dose of auraptene 4 µM was evaluated as a control.

### 2.13. Isolation of EVs from Cell Culture-Conditioned Medium

After treatments, β-cells conditioned medium was diluted in PBS and centrifuged twice at 200× *g* for 15 min using an S50-A 2559 rotor; subsequently, the pellet was discarded, and the supernatant was recovered. In the next step, the supernatant was centrifuged at 600× *g* for 30 min, the pelleted cell debris was discarded, then the supernatant was centrifuged at 2000× *g* for 30 min, later at 10,000× *g* for another 30 min, and finally at 125,000× *g* for 80 min in a Sorvall MTX 150 ultracentrifuge. This ultracentrifugation step generates EVs’ pellet [34]. The pellet was suspended in PBS and centrifuged one last time to eliminate contaminant proteins, mainly insulin, and obtain a fraction with a high degree of purity. The quality of the isolated EVs was characterized by the markers flotillin-2 and CD63.

### 2.14. Extracellular Vesicle Analysis

C9 cells were grown on 20-mm well plates (100,000 cells/mL) in DMEM supplemented medium. At 90% confluence, C9 cells were incubated under different treatments with 40 μL of EVs isolated from β-cells previously incubated under several conditions. Afterward, the cells were washed with ice-cold PBS and lysed for 35 min at 4 °C with protein-lysis buffer and centrifuged for 10 min at 8000 rpm. The supernatant was recovered, and protein was quantified using a BCA assay. Samples (25 µg) were electrophoresed on SDS-PAGE (10%) gels and transferred to PVDF membranes to evaluate the protein targets of insulin signaling mTOR/p70S6Kα, as well as cholesterol targets.

### 2.15. MTT Assay

Cell viability was assessed by MTT reduction assay under different conditions for 20 h. First, cells were seeded onto 96-well plates at 23,000 cells/well density and allowed to grow at 90% of confluence. Next, the culture medium was replaced with an Opti-MEM medium. After 2 h under this condition, cells were treated under several experiments. Later, 30 μL of an MTT 2.1 mg/mL stock solution was added to the culture media to obtain a final concentration of 0.5 mg/mL. Formazan crystals formed after 4 h of incubation were further dissolved by adding buffer lysis (20% sodium dodecyl sulfate, 50% N, N-dimethylformamide, pH 4.0), according to the previous report [29]. Finally, optical density was measured at 570 nm using a microplate reader.

### 2.16. Organelle Fractionation

Cells grown in 100 mm dishes were treated with LDL (25 µg/mL) for 20 h. Later, cell cultures were washed once with cold PBS and scrapped. Cells were homogenized in 1 mL of buffer 0.27 M sucrose and 10 mM MOPS-Tris (pH 6.8) (SMT buffer), passing the cell suspension three times through a 21-gauge needle and a 27-gauge needle. Cell debris and nuclei were removed by centrifugation for 5 min at 1000× *g*. The supernatant was recovered, and the pellet was homogenized in 0.25 mL of SMT buffer by passing them three times through a 21-gauge needle and a 27-gauge needle, centrifuged for 5 min at 1000× *g*; the resulting supernatant was pooled with the first one. Pellet corresponds to the nucleus, and the supernatant was centrifuged at 25,000× *g* for 20 min to separate organelles from the cytosol. The pellet was resuspended in 150 µL SMT, loaded on a discontinuous Nycodenz gradient composed of three layers (23.4, 8.8, and 4.4%), and centrifuged at 107,000× *g* for 75 min. The Nycodenz-enriched fraction was recovered and loaded on a 27% Percoll solution, later centrifuged at 35,000× *g* for 45 min.

### 2.17. Molecular Docking Experimentation

Atomic coordinates of protein ACAT/SOAT (PDB ID: 6L47) with a resolution of 3.50 Å were evaluated. The structure of auraptene (CID: 1550607) was obtained from the PubChem database [35]. For docking experimentation, the protein structure was prepared; waters and small molecules were removed. The ligand and protein were 3D-protonated and energy minimization was carried out by employing Molecular Operating Environment software (MOE) using default parameters (Placement: Triangle Matcher, Rescoring 1: London ΔG, AMBER99 force field). Each ligand was generated up to different conformations and protein was visualized with ligand interactions implemented in MOE.

### 2.18. Statistical Analysis

Data are expressed as mean ± SD. Statistical analyses were conducted with one-way ANOVA, and differences among means were compared with the Bonferroni assay using a significance level of *p* < 0.01, unless otherwise specified. The software used was GraphPad Prism version 6 (San Diego, CA, USA). For the analysis of the expression of proteins by Western blot, a semiquantitative analysis was performed using loading controls with the ImageJ software (Bethesda, MD, USA).

## 3. Results

### 3.1. Characterization of LDL Internalization in Insulin-Secreting Cells and Expression of Cholesterol Protein Targets

**Figure 1:**
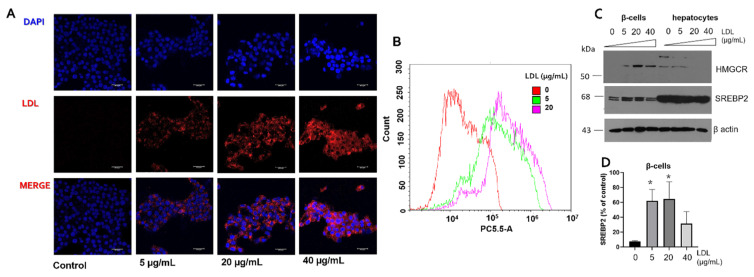
LDL endocytosis in β-cells is associated with the alteration of cholesterol targets. (**A**) RIN-m5F cells were treated with increasing concentrations of dil-LDL (0–40 µg/mL), representative images showing Hoescht (blue), dil-LDL (red), and merge for each treatment. (**B**) Flow cytometry analysis of dil-LDL endocytosis (0–20 µg/mL), under incubation for 20 h. PBS was used as a vehicle. (**C**) Effect of LDL treatment (0–40 µg/mL) on the expression of HMGCR and SREBP2 in β-cells, hepatocytes were evaluated as a control. (**D**) Densitometry analysis of SREBP2 in β-cells, results are reported as mean ± SD (*n* = 3), * *p* < 0.05 concerning control. β-actin was used as a loading control.

We focused on the effect of LDL treatment on the cholesterol management capability of β-cells. In the first instance, LDL isolation was performed by the KBr ultracentrifugation method and validated through the evaluation of protein targets apoA-1 and apoB-100 on the VLDL, LDL, and HDL fractions. Our results verified the appropriate isolation of the LDL fraction (Supplementary Figure S1). According to previous work, the LDL fraction was labeled with a fluorescent dil-C18 probe (dil), obtaining dil-LDL particles [36]. β-cell cultures were treated under a concentration range of dil-LDL (0–40 µg/mL) (Figure 1A). Based on our previous work associated with the fatty acid overload and lipopolysaccharide effect on β-cell proteostasis and its impact on insulin secretion [17], we have established a critical range in the β-cell physiology, the interval of 18–24 h, in which biological phenomena such as changes in gene expression occur, with an insulin half-life of approximately 30 h [37]. Therefore, as a criterion for acute stimuli, we selected a range of 20 h. Results suggest a concentration-dependent dil-LDL internalization, wherein the dil-LDL signal was located in the cytoplasm and perinuclear area (Figure 1A). In parallel, LDL characterization was accomplished by flow cytometry; results confirmed the LDL endocytosis phenomenon (Figure 1B). As a control, we characterized the LDL endocytosis on hepatocyte cultures; dose-dependent (0–40 µg/mL) endocytosis was registered by confocal microscopy, finding a similar response in the LDL internalization (Supplementary Figure S2).

To gain insights into the mechanisms involved in cholesterol regulation under LDL treatment, we characterized the expression of HMGCR and SREBP2, critical elements for the de novo cholesterol pathway. The data suggest a specific regulation of these targets in β-cells and hepatocytes, wherein cholesterol synthesis could be triggered in β-cells, considering an increased SREBP2 and HMCGR expression (Figure 1C,D). This response may suggest an abnormal cholesterol metabolism in β-cells associated with the internalization and activation of de novo synthesis. In this sense, hepatocytes cultures were evaluated as controls, showing a constant high expression of SREBP2 and an HMGCR reduction (Figure 1C), suggesting a better balance between endocytosis and de novo synthesis of cholesterol. Moreover, a connection between cholesterol homeostasis and Ca^2+^ has been proposed; for instance, a critical condition of the basal sensitivity of the sterol sensing SREBP activity is the concentration of ER Ca^2+^ [18].

### 3.2. Insulin Secretion Is Promoted by LDL Endocytosis and Regulation of Targets That Modulate Intracellular Calcium

**Figure 2:**
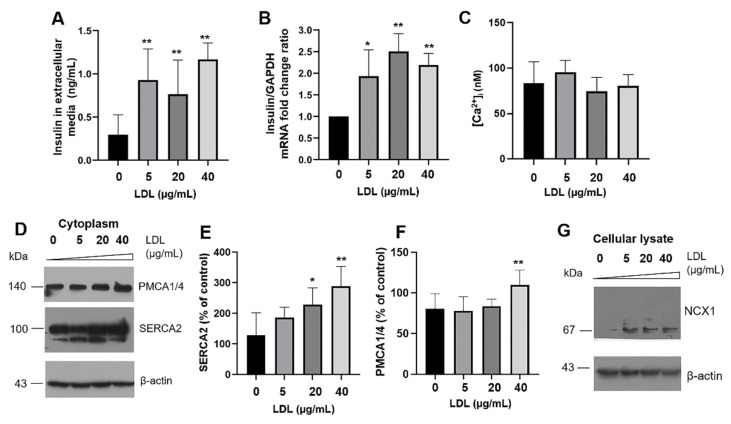
Insulin secretion promoted by LDL is associated with the modulation of targets controlling intracellular calcium levels. (**A**) Effect of LDL treatments (0–40 µg/mL) on insulin secretion (*n* = 3, mean ± SD), ** *p* < 0.005. (**B**) Evaluation of insulin mRNA expression under the same LDL concentrations and 20 h of treatment, qPCR reactions were performed for triplicate, and GAPDH was used as a reference calibrator. Results are reported as mean ± SD (*n* = 3), * *p* < 0.05, ** *p* < 0.005. (**C**) Quantification of intracellular Ca^2+^. (**D**) Expression of PMCA1/4 and SERCA2 on cytoplasm lysates, representative Western blots are showed; quantitative characterization of the (**E**) SERCA2 and (**F**) PMCA1/4 expression (*n* = 3, mean ± SD), * *p* < 0.05, ** *p* < 0.005. (**G**) Western blot of NCX1 in cellular lysates; β-actin was used as a loading control.

Considering the presence of LDL endocytosis in β-cells and its potential implications on regulating metabolism by modifying insulin secretion, we explored the secretagogue effect of LDL and the possible dependence on Ca^2+^ concentrations. Contrary to expectations, the results showed increased insulin exocytosis activity under the LDL incubation in a concentration-dependent manner (0–40 µg/mL), evaluated by the measurement of insulin concentrations in the extracellular media (Figure 2A).

To gain further insights into the cellular responses originated by LDL treatment, we characterized the insulin mRNA levels, wherein an increased expression was detected (Figure 2B), with behavior corresponding to the augmented protein concentrations in the extracellular media. Our data suggest the presence of a hyperinsulinism response mediated by LDL. Notwithstanding, intracellular calcium concentrations were maintained without significant changes at basal levels (Figure 2C); in this case, we evaluated the calcium concentrations by obtaining for each measurement the *Rmax* and *Rmin* parameters [33]. To explain this phenomenon, we focused on the characterization of primary regulators of cytoplasmic calcium concentrations. Our results showed the overexpression of PMCA1/4 and SERCA2 in cytoplasmic fractions of β-cells dependent on LDL treatment (Figure 2D–F). In addition, we broadened the description of these cellular responses, exploring the LDL effect on the Na^+^/Ca^2+^ exchanger NCX-1 (Figure 2F,G). Critically, the increase of PMCA1/4, SERCA2, and NCX-1 could play an adaptive response to modulate cytoplasmic calcium and then insulin exocytosis. In contrast, in a previous report, saturated palmitic acid in combination with lipopolysaccharides promoted the proteolytic degradation of PMCA1/4, affecting the regulation of calcium, insulin exocytosis, and cellular viability [17].

**Figure 3:**
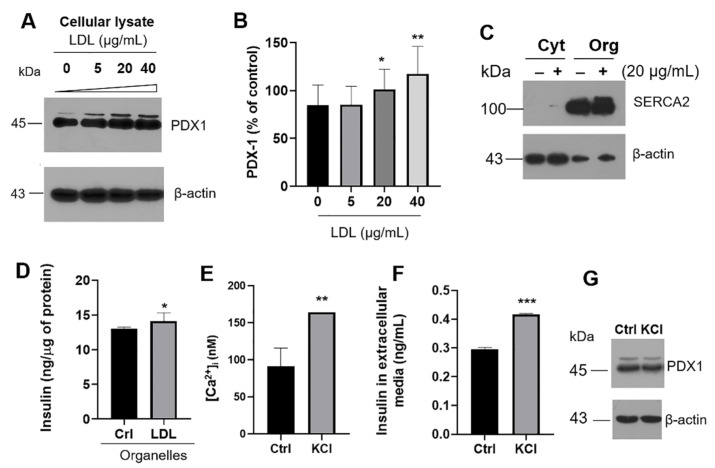
LDL modulates the extracellular insulin concentration through a transcriptional mechanism. (A) PDX-1 expression under LDL treatment (0–40 µg/mL). (B) Densitometry analysis of PDX-1 in β-cells, results are reported as mean ± SD (*n* = 3); * *p* < 0.05, ** *p* < 0.005 with respect to control. (C) Characterization of SERCA2 on cytosol (Cyt) and organelles (Org) isolates; for this experimentation, LDL (20 µg/mL) effect was evaluated. (D) Under this condition, insulin quantification was performed in organelles fraction; effect of KCl depolarization (30 mM) on intracellular Ca^2+^ concentration (E) and insulin in extracellular media (F). Stimuli were performed for 40 min. (**D**–**F**) Results are reported as mean ± SD (*n* = 3), * *p* < 0.05, ** *p* < 0.01, *** *p* < 0.005. (G) Under the same condition, PDX-1 expression was characterized. In panels (**A**,**C**,**G**) β-actin was used as a loading control.

To explore into the mechanisms inducing the increment in LDL-dependent insulin secretion, the expression of pancreatic and duodenal homeobox 1 (PDX-1) was evaluated. PDX-1 is the critical transcription factor for insulin expression and, with the gene BETA2, is associated with β-cell proliferation [38]. LDL treatment (0–40 µg/mL) induced an increase in the levels of PDX-1 (Figure 3A,B). To assess whether insulin mRNA and extracellular-insulin levels correlated with an increase in the intracellular concentration, we performed two methodologies for the isolation of several organelles. We showed the results obtained in cytosolic and organelles fractions, where targets such as ER-SERCA2 were evaluated as a control (Figure 3C). The insulin quantification in organelles suggests that LDL treatment (20 µg/mL) promoted an increment (Figure 3D). This phenomenon could explain the effects registered in the insulin mRNA levels and secretion; therefore, the impact of cholesterol overload could be mediated through PDX-1.

As a control, we evaluated the effect of the main secretagogue that regulates intracellular calcium, K^+^, the classical inducer of membrane depolarization (Figure 3E,F). β-cells were stimulated with KCl (30 mM) for 40 min, according to Scullion et al., 2012. Under this condition, results confirm the increase of Ca^2+^ levels induced by KCl (Figure 3E), corresponding with insulin levels in extracellular media (Figure 3F) and evidencing the changes in the electrochemical gradient in the membrane. However, PDX-1 expression levels remained constant under this condition (Figure 3G). Then, cholesterol overload must be the factor that turns on the expression of PDX-1, and a predominant LDL-metabolic effect on the insulin transcription is present, possibly accompanied by lipotoxicity.

### 3.3. Evaluation of UPR Activation and Inflammatory Pathway

**Figure 4:**
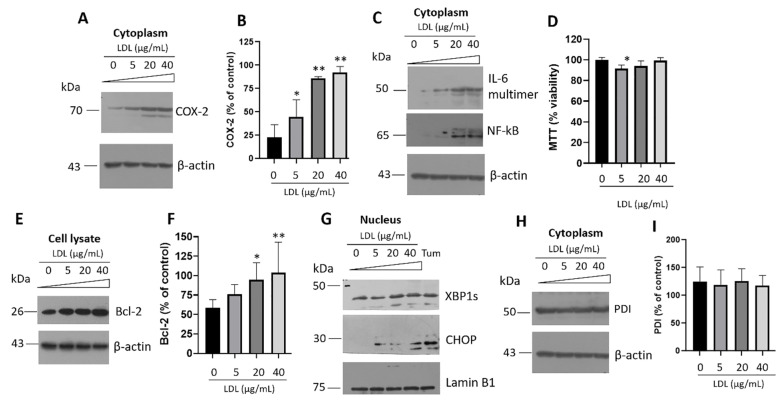
In β-cells, activation of UPR induced by LDL is connected with inflammatory markers. (A) COX-2 expression under the LDL treatment (0–40 µg/mL) for 20 h in cytoplasm. Tunicamycin (Tum, 1 µg/mL) was used as an ER-stress inducer. (B) Densitometry analysis of COX-2, results are reported as mean ± SD (*n* = 3) and expressed as % of control, * *p* < 0.1, ** *p* < 0.0005. (C) IL-6 and NF-κB expression under LDL increasing concentrations. (D) Effect of LDL treatment (0–40 µg/mL) on β-cell viability, results are reported as mean ± SD (*n* = 6), * *p* < 0.05. (E) Under the same conditions, expression of apoptosis regulator, Bcl-2; (F) quantitative characterization of the Bcl-2 expression (*n* = 3, mean ± SD), * *p* < 0.05, ** *p* < 0.005. (G) Western blot of the UPR targets XBP1s and CHOP under LDL stimuli (0–40 µg/mL) in nucleus isolates. Lamin B1 was used as a loading control. Tunicamycin (Tum) was used as a control (1 µg/mL). (H) PDI expression levels under the LDL treatment. (I) Densitometry analysis of PDI, results are reported as mean ± SD (*n* = 3) and expressed as % of control. In panels A, C, E, and H, β-actin was used as a loading control.

We previously demonstrated that lipotoxicity induced cyclooxygenase-2 (COX-2) overexpression in hepatocytes, connected with steatosis, activating PERK/CHOP signaling [39]. In β-cells, results suggested that hypercholesterolemic LDL-stimuli was coupled with the induction of COX-2 expression (Figure 4A,B). Moreover, an associated inflammatory cell response was registered by the IL-6 induction dependent on NF-κB expression (Figure 4C). However, exposure to high LDL concentration was not enough to modify the cellular viability (Figure 4D). In this regard, we registered a pro-survival response mediated by LDL, evaluating anti-apoptotic Bcl-2 (Figure 4E,F), possibly generating a compensatory mechanism. Data suggest the evidence of a pro-inflammatory response without affecting cellular viability. These results support the low-grade systemic inflammation connected with obesity [40], considering obese patients showed increased levels of plasmatic IL-6 and IL-1β, inducing a higher risk of β-cell dysfunction and T2DM [41]. For the first time, this phenomenon is described in β-cells.

Chronic hyperglycemia and hyperlipidemia associated with T2DM disrupt ER homeostasis, which could originate the UPR activation [42]. LDL treatment promoted the translocation of the transcriptional factors XBP1s and CHOP in the nucleus, indicative of the activation of the IRE1 and PERK arms of UPR (Figure 4G). Tunicamycin (Tum), an ER-stress inducer that inhibits N-linked glycosylation, was included as a control (Figure 4G). The expression of genes involved in lipid synthesis and chaperones could accompany this response to alleviate ER stress [42], a phenomenon confirmed by the modulation of the lipid synthesis regulator SREBP2, as we demonstrated in Figure 1. Misbalances in the secretory protein synthesis pathway could be critical during insulin maturation; proinsulin is folded at the ER by chaperones such as protein disulfide isomerase (PDI) [43]. The PDI response was constant under LDL treatment (Figure 4H,I), suggesting the importance of maintaining insulin folding. Furthermore, we completed the PDI characterization on ER isolates showing the same response (Supplementary Figure S3). We have described the importance of PDI under proteotoxicity induced by islet amyloid polypeptide (IAPP)-derived peptides under β-sheet conformation [44]; IAPP is a hormone co-secreted with insulin. Described as a chaperone with critical functions, PDI has a high expression level in the ER lumen, to a lower extent in the cytosol and different cellular membranes [45]. Taking into account the high PDI expression and according to these results and previous characterization, we suggest that maintaining a high PDI expression would be critical in β-cell homeostasis. Likewise, despite the over-activation of pro-inflammatory proteins and the UPR, β-cell viability was not affected under LDL stimuli (Figure 4D). Although, high levels of LDL and the exacerbated cellular disposal of cholesterol could affect other sensitive mechanisms.

### 3.4. Effect of LDL Treatment on Renin–Angiotensin System (RAS) Components

**Figure 5:**
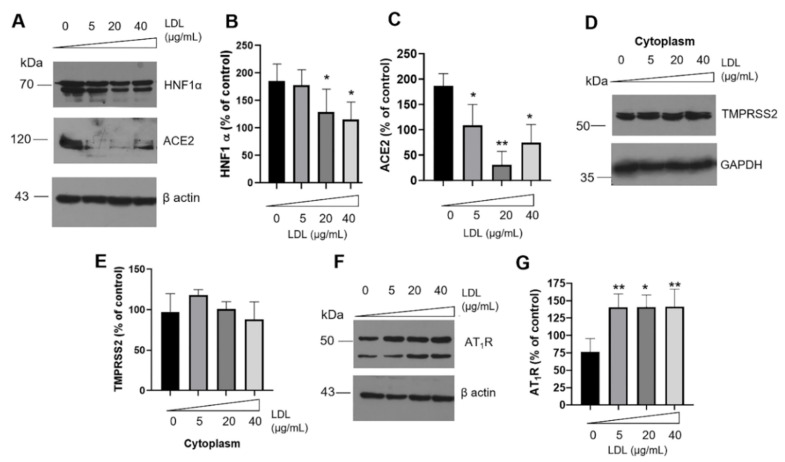
Effect of LDL treatment on renin–angiotensin system (RAS). (**A**) Western blot of HNF1α and ACE2 under increasing concentrations of LDL (0–40 µg/mL) at 20 h treatment. Quantitative characterization of HNF1α (**B**) and ACE2 (**C**), results are expressed as % of control, * *p* < 0.05, ** *p* < 0.005 (*n* = 3, mean ± SD). (**D**) Under the same experimental condition, expression of TMPRSS2 on cytoplasm extracts; (**E**) densitometry analysis of TMPRSS2 (*n* = 3, mean ± SD). GAPDH was used as a loading control. (**F**) AT_1_R expression levels under LDL incubation. (**G**) Densitometry analysis of AT_1_R, results are expressed as % of control, * *p* < 0.05, **p < 0.005 (*n* = 3, mean ± SD). In panels (**A**,**F**), β-actin was used as a loading control.

An impaired glucose tolerance and insulin resistance are associated with ACE2-knockout [46,47], and possibly, HNF1α plays a critical role. Under LDL treatment in β-cells, we registered a distinctive LDL-dependent drop in HNF1α (Figure 5A,B). Expression levels of HNF1α correspond with ACE2 levels (Figure 5A–C), a consistent phenomenon since HNF1α has been described as the primary regulator of ACE2 [22], without affecting the synthesis of insulin, whose main transcription factor is PDX-1. Specific mechanisms whereby LDL modulates HNF1α expression are not elucidated. Taking into account that the expression of cholesterol regulators such as PCSK9 is controlled mainly at the transcriptional gene level by HNF1α [48], intracellular cholesterol overload [49] and the induction of ER stress associated with lipotoxicity could disrupt HNF1α expression.

To elucidate the potential implication of LDL stimulus on RAS, we focused on the ACE2 primary regulator, the transmembrane Serine Protease 2 (TMPRSS2) (Figure 5D). In the first instance, we reported the expression of TMPRSS2 in β-cells, as has been reported by other study [50]. Under LDL stimulus, results suggested the presence of basal levels of TMPRSS2 in the cytoplasmic fractions (Figure 5D). Although ACE2 and HNF1α diminished, the expression of TMPRSS2, a regulator in binding to various ligands such as modified LDL, remained constant (Figure 5D,E). Indeed, in a meta-analysis, the co-regulation of ACE2 and TMPRSS2 has been suggested [51].

Cellular responses triggered by the downregulation of HNF1α/ACE2 were broadened; in this case, the increased expression level of AT_1_R dependent on the LDL concentration was registered (Figure 5F,G), possibly as an initial cascade of events during metainflammation and RAS hyperactivity. Evidence suggests that chronic hypercholesterolemia induced the upregulation of the AT_1_R function in vivo [41]. Under our conditions, AT_1_R expression was promoted by high LDL concentrations, being described for the first time in β-cells as part of the metainflammatory response. According to meta-analysis, AT_1_R blockers could reduce the risk for new-onset T2DM in individuals with hypertension or an elevated cardiovascular risk [42]. However, their impact on the physiology of β-cells has not been proposed. Since insulin maturation occurs at the ER and Golgi lumen and UPR activation reduces ER stress, cholesterol storage alterations could have critical implications and impair mechanisms such as the RAS.

### 3.5. Intracellular Cholesterol Accumulation Induces Transporter Expression

**Figure 6:**
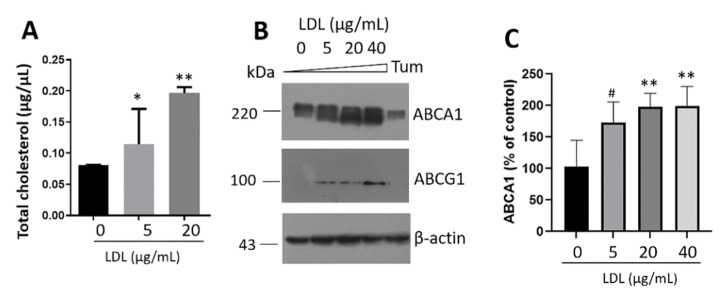
LDL treatment promotes the intracellular accumulation of cholesterol and triggers ABCA1 and ABCG1 expression. Intracellular cholesterol concentrations (**A**) on fat droplets under treatment with LDL (0–20 µg/mL) at 20 h. Results are reported as mean ± SD (*n* = 3), * *p* < 0.1, ** *p* < 0.005. (B) Western blot of cholesterol transporters ABCA1 and ABCG1. β-actin was used as a loading control. Tunicamycin (Tum) was used as a control (1 µg/mL). (C) Densitometry analysis of ABCA1, results are reported as mean ± SD and expressed as % of control (*n* = 3), # *p* < 0.05, ** *p* < 0.005.

To gain insights into the intracellular cholesterol responses under treatment with LDL (0–20 µg/mL), we isolated the fat droplet fraction of β-cells according to the procedure reported by Harris et al., 2012 [30]. We identified a sustained increase in cholesterol concentrations (Figure 6A); this response was coupled with an overexpression of the transporters ABCA1 and ABCG1 (Figure 6B,C) as an adaptive mechanism triggered under the cholesterol accumulation. Cellular cholesterol homeostasis is controlled by the uptake, synthesis, and efflux. Cholesterol efflux is critical in several conditions, for instance, reverse-cholesterol transport [51], and possibly in the physiology of highly metabolic β-cells. Therefore, the overexpression of cholesterol transporters is a compensatory mechanism to maintain homeostasis. ER stress promoted by Tum did not induce ABCA1 overexpression (Figure 6B), suggesting a metabolic response to the ABCA1.

Results suggest that LDL treatment modified the intracellular cholesterol metabolism, coupled with alterations of insulin secretion and signaling. Under metainflammation, the accumulation of bioactive lipids such as diacylglycerols as a response to triglyceride biosynthesis triggers protein kinase C-ε activation to disrupt insulin signaling [52]. Then, metabolic tissues could be susceptible to intracellular lipid modifications. In this sense, acyl-coenzyme A: cholesterol acyltransferase (ACAT1), is an integral protein of ER, catalyzing cholesteryl esters (CE) synthesis from cholesterol and fatty acyl-coenzyme A. CE are stored as cytoplasmic lipid droplets inside the cells [53,54] and are associated with insulin metabolism. ACAT1 activity could impact ABCA1 cholesterol export, considering ABCA1 mediates the removal of free cholesterol and phospholipids from lipid-laden cells [55]. We have, therefore, sought the regulation of metainflammation through multifaceted approaches, for instance, by exploiting the broad diversity of natural compounds found in plants [39] with, mainly, dietary phytochemicals represent a promising therapy.

### 3.6. Regulation of Intracellular Cholesterol Metabolism by Auraptene

**Figure 7:**
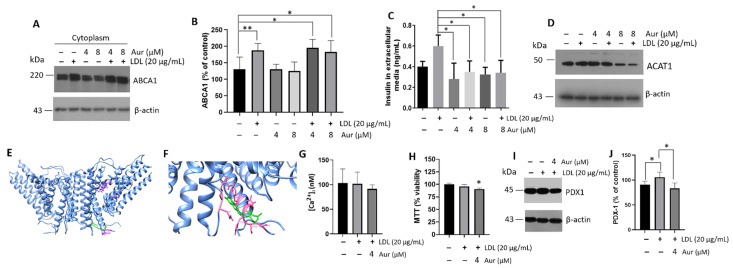
Auraptene (Aur) treatment modulates insulin secretion through intracellular cholesterol metabolism in β-cells. (A) Characterization of ABCA1 under LDL (20 µg/mL) and concomitant treatment with Aur (4 and 8 µM) at 20 h, control treatments were performed with Aur. (B) Densitometry analysis of ABCA1, results are reported as mean ± SD (*n* = 3) and expressed as % of control, * *p* < 0.05, ** *p* < 0.005. (C) Effect of LDL (20 µg/mL) and Aur treatment (0–8 µM) on insulin secretion in extracellular media (*n* = 3, mean ± SD), * *p* < 0.05 with respect to LDL treatment. (D) Under the same conditions, with ACAT1 expression in cellular lysates, β-actin was used as a loading control. DMSO was evaluated as a control. (E,F) Representation of the ACAT1 (blue, PDB: 6L47) binding to Aur (green) by molecular docking; the residues (pink) indicate the interactions with Aur. (G) Intracellular calcium quantification under LDL (20 µg/mL) and Aur treatment (4 µM) at 20 h of incubation; a statistical significance was not found. Under the same conditions, the cellular viability (H) and expression of PDX-1 by Western blot (I) were evaluated. (J) Densitometry analysis of PDX-1, results are reported as mean ± SD and expressed as % of control (*n* = 3), * *p* < 0.05. In panels (**A**,**D**,**I**) β-actin was used as a loading control.

We focused on strategies to inhibit cholesterol esterification and thus prevent its accumulation, inducing mechanisms that carry out cholesterol to the extracellular media through the transporter ABCA1. ACAT can be regulated by an Aur molecule [28], a geranyloxyn coumarin found in citrus fruits and *Ferula* species [56]. Based on this evidence and the activity of cholesterol transporters, we focused on the effect of Aur on ABCA1 expression. First, we determined that the ABCA1 compensatory mechanism mediated by LDL is maintained under Aur treatment (0–16 µM) (Supplementary Figure S4). Then, we focused on the Aur effect at 4 and 8 µM, and the concomitant LDL treatment with Aur maintained an elevated expression of ABCA1 (Figure 7A,B). This phenomenon was coupled with regulating the hyperinsulinism promoted by LDL treatment; remarkably, Aur reduced the insulin peak induced by LDL (Figure 7C). This may be associated with the inhibition of ACAT and, possibly, the induction of its degradation (Figure 7D). A dynamic balance between the esterification and hydrolysis of cholesterol in cells could be essential to maintain homeostasis [57], considering that the esterification of sterols and hydrolysis of cholesteryl-esters could buffer both an excess and a deficiency of free sterols [58].

In addition, by employing *in silico* experimentation on an ACAT structure (PDB: 6L47), molecular docking results suggest the interaction of Aur with the residues Arg_350_, Asn_351_, Tyr_523_, Arg_525_, Gln_526_, His_527_, Cys_528_, and Pro_529_ (Figure 7E,F). The Aur molecule could move away from the active site of ACAT when exposed to the solvent, which does not allow the interaction of Aur with the crucial catalytic residues within the active site (H_460_, N_421_, and W_420_) [59]. The crystal structure of ACAT1 reveals a tetramer integrated by a dimer with a symmetric rubber raft shape. Each subunit comprises nine transmembrane helices, showing a disulfide bond (C_528_ and C_546_) that is not modified by the interaction with Aur [59,60]. An inhibitory phenomenon on cholesterol esterification could occur, inducing higher levels of free cholesterol for cellular exportation and impacting the ABCA1 activity (Figure 7). Strategies such as ACAT inhibition showed relevant functions regulating the β-cell physiology. Under the concentration evaluated (4 µM), Aur did not affect the intracellular calcium levels (Figure 7G) and only a subtle difference in cellular viability concerning the controls was registered (Figure 7H). Moreover, we have shown the cellular effect of Aur in a range of 4–16 µM on the expression of ABCA1. However, we have restricted ourselves to the lowest Aur 4 µM concentration. Our data suggest Aur modulates PDX-1 activation under the LDL treatment (Figure 7I,J), showing that Aur induces the regulation of insulin secretion by PDX-1. The results point out the role of Aur in metabolism regulation [61].

The regulation of the PERK arm of UPR in β-cells could prevent liver steatosis in mice under a high-fat diet [5] and, possibly, a communication process parallel to insulin among these tissues could be mediated by EVs. Furthermore, once in circulation, EVs can regulate intercellular communication in an endocrine way, facilitating the transmission of reciprocal signals among different tissues involved in metabolism [62]. Therefore, metainflammation in β-cells could modify cholesterol metabolism and promote insulin resistance in distant tissues via EVs.

### 3.7. Cellular Communication Mediated by EVs

**Figure 8:**
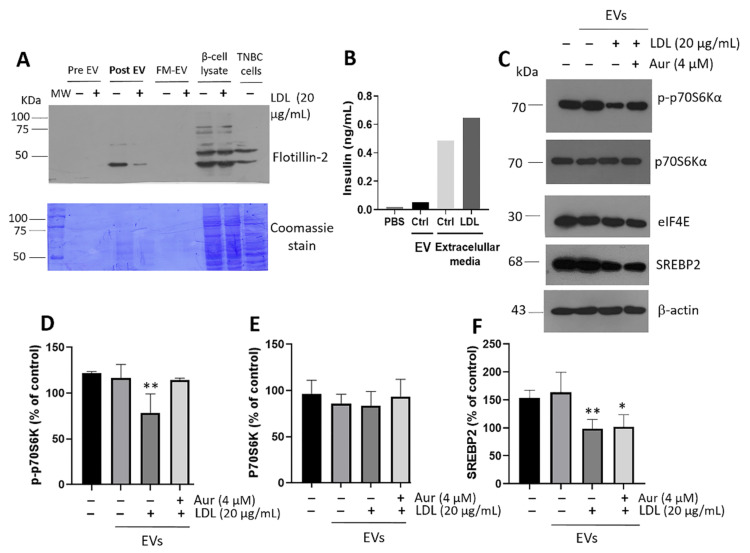
The effect of extracellular vesicles (EVs) in β-cells on insulin signaling in hepatocytes. (A) Characterization of the purity of EVs isolated in extracellular media of β-cells was carried out through several steps; flotillin-2 was used as a marker. Pre-EVs: extracellular vesicles at 0 h; Post-EVs: extracellular vesicles at 20 h; FM-EVs: free medium of extracellular vesicles. β-cells and triple-negative breast cancer cells (TNBC) lysates were used as controls. In addition, Coomassie stain was performed on PVDF membranes. (B) Insulin quantification in EVs samples and extracellular media of β-cells employing the ultrasensitive insulin ELISA assay. (C) Characterization of insulin signaling in hepatocytes by p-p70S6kα, p70S6kα, eIF4E, and SREBP2 under the treatment of EVs derived from β-cells incubated with LDL (20 µg/mL) and concomitant Aur (4 µM) treatment, respectively. PBS was used as a control. Densitometry analysis of p-p70S6kα (D), p70S6kα (E), and SREBP2 (F). Results are reported as mean ± SD (*n* = 3) and expressed as % of control, * *p* < 0.05, ** *p* < 0.01.

We evaluated this proposal employing EVs released under LDL treatment. First, β-cells were incubated for 24 h with LDL (20 µg/mL) and concomitant Aur (4 µM); then, treatments were withdrawn and cells were washed and incubated for another 24 h. Therefore, we ruled out the background effect of LDL. Next, the supernatant medium was recovered and the isolation of the EVs fraction was performed. In the first instance, we confirmed a correct EVs isolation process by characterizing the exosome marker flotillin-2 on several extracellular media taken at different experiment stages. The results showed that adequate EVs fractions could be obtained 24 h after LDL treatment (Figure 8A). Likewise, the isolation process demonstrated the presence of irrelevant insulin concentration in EVs fractions, thus ruling out interferences (Figure 8B). As a criterion of reference, the insulin in EVs was compared with concentrations in the supernatant media of control and LDL-treated RIN-m5F cells (Figure 8B). In a complementary way, VPS4 protein was evaluated in β-cells, a target associated with the endosomal sorting complexes required for transport (ESCRT) machinery and essential for EVs formation [63]. VPS4 expression levels were evaluated under concomitant LDL and Aur treatment and significant changes were not registered (Supplementary Figure S5), suggesting the EVs formation is not affected.

The effect of EVs isolated from β-cells was characterized under the treatment of hepatocytes cultures for 20 h. We found a diminished insulin signaling phenomenon under the EVs incubation in which vesicles were isolated from β-cells treated with LDL; this phenomenon was characterized by a reduction in the activation of p-p70S6Kα, which was dependent on mTOR signaling, without affecting the expression levels of p70S6Kα (Figure 8C–E). p70S6Kα activation is a crucial target in the regulation of protein translation, cellular growth, and proliferation, dependent on insulin signaling [64]. Notably, the concomitant treatment of LDL plus Aur (4 µM) avoided this reduction in the p-p70S6kα (Figure 8C–E). Direct treatment with LDL (20 µg/mL) and insulin (28 IU/L) was used as a control (Supplementary Figure S6). Protein translation modifications have been described in obesity, promoting the translation of targets that modulate lipid accumulation. A determining factor is the cap-binding eukaryotic translation initiation factor 4E (eIF4E) [65], a hub target in protein translation and lipogenesis for which regulation depends on Akt/mTOR (Figure 8C). Moreover, the EV-induced cellular alteration was coupled with a modification in the expression of the SREBP2 transcription factor (Figure 8C,F). The results confirm the affectation induced by EVs isolated from LDL-treated cells.

## 4. Discussion

Based on the characterization of insulin phenomena such as exocytosis and mRNA synthesis, β-cells were highly sensitive to LDL. Therefore, compensatory mechanisms mediated by ABCA1 and calcium-regulator proteins were triggered. Calcium management through the overregulation of SERCA2, NCX1, and PMCA1/4 could maintain free Ca^2+^ at a resting concentration of ∼100 nM [66]. Enzymes in charge of cholesterol metabolism, calcium homeostasis, and ones involved in regulating plasmatic membrane cell turnover and dynamics are located in the ER. UPR is a crucial regulator of the metabolism of lipids and sterols [67]. Thus, a direct connection between ER stress and cholesterol overload could be established, with consequences on calcium regulation, insulin exocytosis, and the alteration of RAS.

Based on our results, a cellular response promoted by high LDL concentrations was the activation of UPR; in this regard, under a saturated lipid stimulus, the UPR sensitization of β-cells by IRE1/XBP1s and PERK/CHOP contributed to the upregulation of inflammatory pathways mediated by NF-κB [17,68], impairing insulin synthesis and glucose-stimulated insulin secretion (GSIS) [27]. Our evidence suggests that the induction of an adaptive mechanism, triggered by LDL-cholesterol overload, and slight UPR activation promote a hyperinsulinism phenomenon correlated to the synthesis of proinflammatory cytokines.

Therefore, the simultaneous function of Ca^2+^ management proteins and UPR maintains an essential contribution to the regulation of insulin exocytosis. Subtle mechanisms to sustain cholesterol homeostasis involve esterification and hydrolysis [57]; cholesterol biosynthesis is performed in several organelles, ER acts as the primary site of biosynthesis, and enzymes such as HMG-CoA reductase reside and control the rate-limiting synthesis. Under LDL treatment, β-cells increased the expression of HMG-CoA reductase and SREBP2 (Figure 1), and an active mechanism for cholesterol management could be present and increase intracellular levels in agreement with LDL stimulation. In addition, a cholesterol-induced inflammatory response occurred in parallel with the hyperinsulinism mediated by the PDX-1 mechanism, impacting the physiology of β-cells. Therefore, based on our results, novel mechanisms induced a hyperinsulinism phenomenon connected with an LDL-cholesterol overload.

LDL treatment increased insulin gene expression; however, intracellular calcium and counterbalance mechanisms might be triggered to maintain β-cell proteostasis. Hyperinsulinism was associated with ER stress, metainflammation, and a compensatory response of ABCA1 overexpression. On the other hand, the highly proatherogenic oxidized LDL has been described as downregulating ABCA1 expression via the MEK/ERK/LXR pathway, leading to impaired insulin synthesis and GSIS in a β-cell model [69]. Indeed, ABCA1 reduction could lead to cholesterol accumulation coupled with an increased risk of islet amyloid polypeptide (IAPP) aggregation [70], which suggests the importance of ABCA1. According to our previous results, lipid overload affects the structural characteristics and stability of IAPP, modifying its secretion [44]; this phenomenon was accompanied by β-cells islet cytotoxicity [44]. In this work, our results suggest another response associated with ABCA1 overexpression, highlighting the importance of maintaining high levels of ABCA1.

Membrane cholesterol content due to hypercholesterolemia has been associated with low insulin secretion and glucose intolerance [71]. Likewise, a connection between elevated serum cholesterol and reduced insulin secretion in β-cell cultures has been reported; this condition was associated with increased neuronal nitric oxide synthase activation [72], but without establishing a specific mechanism. Our data refer to the initial stages of damage during the prediabetes, wherein one of the hallmarks is hyperinsulinemia, and reveals the adaptive mechanisms that β-cells develop to alleviate this phenomenon. Indeed, during prediabetes, elevated levels of small dense LDL-cholesterol (sdLDL-C) and LDL-triglycerides have been registered [73]. Therefore, conditions of LDL-hypercholesterolemia could represent an early damage mechanism during hyperinsulinism.

Auraptene treatment ameliorated hyperinsulinism coupled with high ABCA1 levels, which maintained cholesterol homeostasis; therefore, the activity of cholesterol transport and the inhibition of cholesterol esterification through ACAT1 could play a significant protective role. Cholesterol should be the regulatory factor that induces the expression of the PDX-1, impacting hyperinsulinemia. In this regard, the insulin promoter has been described as SREBPs target [74]; specifically, SREBP-1c, implicated in fatty acid synthesis genes, can repress PDX-1 expression, promoting the inhibition of insulin expression and β-cell failure, a phenomenon induced under a lipotoxic condition [75]. Based on the results, other transcription factors regulated by sterols such as SREBP2 could promote PDX-1 expression Figure 1C,D and Figure 3A,B). A decrease in the cholesterol accumulation by Aur treatment could increase cholesterol export and Langerhans β-cell functionality, reducing the activation of PDX-1 and, thus, insulin secretion (Figure 7C,I). Pharmacologic treatments in development could enhance insulin synthesis and secretion [76,77], however, approaches that consider the role of lipotoxicity are still limited.

Likewise, an intrinsic connection may exist between the cholesterol regulation and carbohydrate metabolism triggered by factors synthesized in β cells. Our results suggest that the EV originated from metabolic damage in β-cells may contribute to insulin resistance in hepatocytes by decreasing mTOR/p70S6Kα activation. We previously demonstrated that exosomes isolated from supernatants of macrophages exposed to lipopolysaccharides caused a metainflammation phenomenon on hepatocytes, promoting a rearrangement in the cholesterol metabolism [39]. Our evidence suggests the impact of lipid-induced inflammatory conditions on β-cells and their influence on hepatocytes mediated by EVs. As part of this communication mechanism among cells and considering the metabolic dysfunction in β-cells, the effect exerted by metabolic overload is not only seen on insulin secretion, but a critical rearrangement of the cholesterol pathways could also occur and represent the trigger condition. As hyperinsulinemia might be related to liver-cholesterol management, and considering the presence of intercellular communication mechanisms mediated by EVs, β-cell activity could exert a regulatory node at the systemic level. However, this phenomenon can be modulated by the natural compound Aur, a small molecule that displays Lipinski’s criteria in pharmacologic development. Additional studies on pancreatic islets to corroborate this phenomenon are necessary.

The implications between RAS and β-cell metainflammation may impact systemic metabolic regulation and other related mechanisms. The role of HNF1s and ACE2 has been proposed in β-cell activity-dependent glucose homeostasis [46,47]. A defective allele of HNF1α in humans has been shown to cause Maturity Onset Diabetes of the Young (MODY) type 3 [78]. Evidence suggests that HNF1α could control the growth and function of β-cells by the gene regulation of targets such as glucose transporter 2, pyruvate kinase, collectrin, hepatocyte growth factor activator, and HNF4α [79]. Our results suggest that high LDL-cholesterol promotes a reduction in HNF1α, which causes a low expression of ACE2, contributing to the impact on processes implicated in metainflammation.

## 5. Conclusions

The regulation of intracellular cholesterol metabolism represents a fundamental target for insulin secretion, and cholesterol must modulate the concentration of the PDX-1 transcription factor. β-cells showed high metabolic activity, wherein a feedback mechanism in cholesterol management could occur, modifying insulin secretion and activating compensatory mechanisms critical in calcium regulation, proteostasis, and cholesterol homeostasis, mechanisms that allow the maintenance of homeostasis and perform adaptive insulin secretory activity. A concomitant response, originated in β-cells and mediated by EVs, could be triggered in a manner involving tissues such as the liver. The results confirm the role of auraptene on the metabolic effect and implication of LDL on β-cell functionality, maintaining cellular homeostasis, and allow the basis for the use of molecules isolated from natural sources or dietary phytochemicals to be established. Therefore, the results emphasize the importance of strategies to reduce plasmatic LDL concentrations.

## Data Availability

The data presented in this study are available in article and Supplementary Materials.

## Supplementary Materials

The following supporting information can be downloaded at: https://www.mdpi.com/article/10.3390/metabo12080754/s1, Supplementary Figure S1: Characterization of fractions of LDL isolation was obtained by the KBr-ultracentrifugation method. Supplementary Figure S2: Evaluation of protocols employed in cellular fractionation on Langerhans β-cell cultures. Supplementary Figure S3: LDL endocytosis in C9 cells (hepatocytes). Supplementary Figure S4: Characterization of endoplasmic reticulum isolates through the evaluation of protein markers PDI, SERCA2 under LDL treatment. Supplementary Figure S5: Auraptene (Aur) treatment (4–16 µM) regulates the expression of cholesterol transporter ABCA1 in Langerhans β-cells. Supplementary Figure S6: VPS4 expression in Langerhans β-cells under LDL and auraptene (Aur) treatment. Supplementary Figure S7: Effect of LDL (20 µg/mL) and insulin (Ins) (28 IU/L) on insulin signaling in hepatocyte cultures.

## References

[1] Reddy P., Lent-Schochet D., Ramakrishnan N., McLaughlin M., Jialal I. (2019). Metabolic syndrome is an inflammatory disorder: A conspiracy between adipose tissue and phagocytes. Clin. Chim. Acta.

[2] Brunham L.R., Kruit J.K., Verchere C.B., Hayden M.R. (2008). Cholesterol in islet dysfunction and type 2 diabetes. J. Clin. Investig..

[3] Lee Y., Hirose H., Ohneda M., Johnson J.H., McGarry J.D., Unger R.H. (1994). Beta-cell lipotoxicity in the pathogenesis of non-insulin-dependent diabetes mellitus of obese rats: Impairment in adipocyte-beta-cell relationships. Proc. Natl. Acad. Sci. USA.

[4] Weyer C., Hanson R.L., Tataranni P.A., Bogardus C., Pratley R.E. (2000). A high fasting plasma insulin concentration predicts type 2 diabetes independent of insulin resistance: Evidence for a pathogenic role of relative hyperinsulinemia. Diabetes.

[5] Yong J., Parekh V.S., Reilly S.M., Nayak J., Chen Z., Lebeaupin C., Jang I., Zhang J., Prakash T.P., Sun H. (2021). Chop/Ddit3 depletion in beta cells alleviates ER stress and corrects hepatic steatosis in mice. Sci. Transl. Med..

[6] Kouvari M., Chrysohoou C., Skoumas J., Pitsavos C., Panagiotakos D.B., Mantzoros C.S., ATTICA Study Investigators (2022). The presence of NAFLD influences the transition of metabolically healthy to metabolically unhealthy obesity and the ten-year cardiovascular disease risk: A population-based cohort study. Metabolism.

[7] Aso Y., Wakabayashi S., Yamamoto R., Matsutomo R., Takebayashi K., Inukai T. (2005). Metabolic syndrome accompanied by hypercholesterolemia is strongly associated with proinflammatory state and impairment of fibrinolysis in patients with type 2 diabetes: Synergistic effects of plasminogen activator inhibitor-1 and thrombin-activatable fibrinolysis inhibitor. Diabetes Care.

[8] Walter P., Ron D. (2011). The unfolded protein response: From stress pathway to homeostatic regulation. Science.

[9] Scheuner D., Kaufman R.J. (2008). The unfolded protein response: A pathway that links insulin demand with beta-cell failure and diabetes. Endocr. Rev..

[10] Corbett E.F., Michalak K.M., Oikawa K., Johnson S., Campbell I.D., Eggleton P., Kay C., Michalak M. (2000). The conformation of calreticulin is influenced by the endoplasmic reticulum luminal environment. J. Biol. Chem..

[11] Ajoolabady A., Kaplowitz N., Lebeaupin C., Kroemer G., Kaufman R.J., Malhi H., Ren J. (2022). Endoplasmic reticulum stress in liver diseases. Hepatology.

[12] Diaz-Villanueva J.F., Diaz-Molina R., Garcia-Gonzalez V. (2015). Protein Folding and Mechanisms of Proteostasis. Int. J. Mol. Sci..

[13] Chang T.Y., Chang C.C., Ohgami N., Yamauchi Y. (2006). Cholesterol sensing, trafficking, and esterification. Annu. Rev. Cell Dev. Biol..

[14] Axmann M., Strobl W.M., Plochberger B., Stangl H. (2019). Cholesterol transfer at the plasma membrane. Atherosclerosis.

[15] Brunham L.R., Kruit J.K., Pape T.D., Timmins J.M., Reuwer A.Q., Vasanji Z., Marsh B.J., Rodrigues B., Johnson J.D., Parks J.S. (2007). Beta-cell ABCA1 influences insulin secretion, glucose homeostasis and response to thiazolidinedione treatment. Nat. Med..

[16] Fryirs M., Barter P.J., Rye K.A. (2009). Cholesterol metabolism and pancreatic beta-cell function. Curr. Opin. Lipidol..

[17] Acosta-Montano P., Rodriguez-Velazquez E., Ibarra-Lopez E., Frayde-Gomez H., Mas-Oliva J., Delgado-Coello B., Rivero I.A., Alatorre-Meda M., Aguilera J., Guevara-Olaya L. (2019). Fatty Acid and Lipopolysaccharide Effect on Beta Cells Proteostasis and its Impact on Insulin Secretion. Cells.

[18] Wang W.A., Agellon L.B., Michalak M. (2018). Endoplasmic reticulum calcium dictates the distribution of intracellular unesterified cholesterol. Cell Calcium.

[19] Marmugi A., Parnis J., Chen X., Carmichael L., Hardy J., Mannan N., Marchetti P., Piemonti L., Bosco D., Johnson P. (2016). Sorcin Links Pancreatic beta-Cell Lipotoxicity to ER Ca2+ Stores. Diabetes.

[20] Chhabra K.H., Chodavarapu H., Lazartigues E. (2013). Angiotensin converting enzyme 2: A new important player in the regulation of glycemia. IUBMB Life.

[21] Dikalov S.I., Nazarewicz R.R. (2013). Angiotensin II-induced production of mitochondrial reactive oxygen species: Potential mechanisms and relevance for cardiovascular disease. Antioxid. Redox Signal..

[22] Pedersen K.B., Chhabra K.H., Nguyen V.K., Xia H., Lazartigues E. (2013). The transcription factor HNF1alpha induces expression of angiotensin-converting enzyme 2 (ACE2) in pancreatic islets from evolutionarily conserved promoter motifs. Biochim. Biophys. Acta.

[23] Galindo-Hernandez O., Villegas-Comonfort S., Candanedo F., Gonzalez-Vazquez M.C., Chavez-Ocana S., Jimenez-Villanueva X., Sierra-Martinez M., Salazar E.P. (2013). Elevated concentration of microvesicles isolated from peripheral blood in breast cancer patients. Arch. Med. Res..

[24] Huang Z., Xu A. (2021). Adipose Extracellular Vesicles in Intercellular and Inter-Organ Crosstalk in Metabolic Health and Diseases. Front. Immunol..

[25] Martinez M.C., Andriantsitohaina R. (2017). Extracellular Vesicles in Metabolic Syndrome. Circ. Res..

[26] Fu Q., Li Y., Jiang H., Shen Z., Gao R., He Y., Liu Y., Xu K., Yang T. (2019). Hepatocytes derived extracellular vesicles from high-fat diet induced obese mice modulate genes expression and proliferation of islet beta cells. Biochem. Biophys. Res. Commun..

[27] Fernandez-Millan E., Guillen C. (2022). Multi-Organ Crosstalk with Endocrine Pancreas: A Focus on How Gut Microbiota Shapes Pancreatic Beta-Cells. Biomolecules.

[28] De Medina P., Genovese S., Paillasse M.R., Mazaheri M., Caze-Subra S., Bystricky K., Curini M., Silvente-Poirot S., Epifano F., Poirot M. (2010). Auraptene is an inhibitor of cholesterol esterification and a modulator of estrogen receptors. Mol. Pharmacol..

[29] Suski J.M., Lebiedzinska M., Wojtala A., Duszynski J., Giorgi C., Pinton P., Wieckowski M.R. (2014). Isolation of plasma membrane-associated membranes from rat liver. Nat. Protoc..

[30] Garcia-Gonzalez V., Mas-Oliva J. (2017). A Novel beta-adaptin/c-Myc Complex Formation Modulated by Oxidative Stress in the Control of the Cell Cycle in Macrophages and its Implication in Atherogenesis. Sci. Rep..

[31] Harris L.L.S., Shew T.M., Skinner J.R., Wolins N.E. (2012). A single centrifugation method for isolating fat droplets from cells and tissues. J. Lipid. Res..

[32] Patel H., Porter R.H., Palmer A.M., Croucher M.J. (2003). Comparison of human recombinant adenosine A2B receptor function assessed by Fluo-3-AM fluorometry and microphysiometry. Br. J. Pharmacol..

[33] Gomes P.A., Bassani R.A., Bassani J.W. (1998). Measuring [Ca2+] with fluorescent indicators: Theoretical approach to the ratio method. Cell Calcium.

[34] Scullion S.M., Gurgul-Convey E., Elsner M., Lenzen S., Flatt P.R., McClenaghan N.H. (2012). Enhancement of homocysteine toxicity to insulin-secreting BRIN-BD11 cells in combination with alloxan. J. Endocrinol..

[35] (2022). PubChem Compound Summary for CID 1550607, Auraptene. https://pubchem.ncbi.nlm.nih.gov/compound/Auraptene.

[36] Damian-Zamacona S., Garcia-Gonzalez V., Avila-Barrientos L.P., Delgado-Coello B., Reyes-Grajeda J.P., Mas-Oliva J. (2018). Cell survival regulation during receptor-mediated endocytosis of chemically-modified lipoproteins associated to the formation of an Amphiphysin 2 (Bin1)/c-Myc complex. Biochem. Biophys. Res. Commun..

[37] Weiss M., Steiner D.F., Philipson L.H. (2000). Insulin Biosynthesis, Secretion, Structure, and Structure-Activity Relationships. MDText.com, Inc., South Dartmouth (MA). https://europepmc.org/article/nbk/nbk279029.

[38] Guo W., Gong Y., Fu Z., Fu J., Sun Y., Ju X., Chang Y., Wang W., Zhu X., Gao B. (2016). The effect of cholesteryl ester transfer protein on pancreatic beta cell dysfunction in mice. Nutr. Metab..

[39] Galindo-Hernandez O., Cordova-Guerrero I., Diaz-Rubio L.J., Pulido-Capiz A., Diaz-Villanueva J.F., Castaneda-Sanchez C.Y., Serafin-Higuera N., Garcia-Gonzalez V. (2019). Protein translation associated to PERK arm is a new target for regulation of metainflammation: A connection with hepatocyte cholesterol. J. Cell Biochem..

[40] Jung B.C., Kang S. (2021). Epigenetic regulation of inflammatory factors in adipose tissue. Biochim. Biophys. Acta. Mol. Cell Biol. Lipids.

[41] Slepchenko K.G., Chen S., Counts G.P., Corbin K.L., Colvin R.A., Nunemaker C.S. (2021). Synchrotron fluorescence imaging of individual mouse beta-cells reveals changes in zinc, calcium, and iron in a model of low-grade inflammation. Metallomics.

[42] Almanza A., Carlesso A., Chintha C., Creedican S., Doultsinos D., Leuzzi B., Luis A., McCarthy N., Montibeller L., More S. (2019). Endoplasmic reticulum stress signalling—From basic mechanisms to clinical applications. FEBS J..

[43] Michiko S., Yoko Shiba E.D.G.Z. (2018). ER Stress, Secretory Granule Biogenesis, and Insulin. Ultimate Guide to Insulin.

[44] Martinez-Navarro I., Diaz-Molina R., Pulido-Capiz A., Mas-Oliva J., Luna-Reyes I., Rodriguez-Velazquez E., Rivero I.A., Ramos-Ibarra M.A., Alatorre-Meda M., Garcia-Gonzalez V. (2020). Lipid Modulation in the Formation of beta-Sheet Structures. Implications for De Novo Design of Human Islet Amyloid Polypeptide and the Impact on beta-Cell Homeostasis. Biomolecules.

[45] Ali Khan H., Mutus B. (2014). Protein disulfide isomerase a multifunctional protein with multiple physiological roles. Front. Chem..

[46] Bindom S.M., Lazartigues E. (2009). The sweeter side of ACE2: Physiological evidence for a role in diabetes. Mol. Cell Endocrinol..

[47] Niu M.J., Yang J.K., Lin S.S., Ji X.J., Guo L.M. (2008). Loss of angiotensin-converting enzyme 2 leads to impaired glucose homeostasis in mice. Endocrine.

[48] Dong B., Li H., Singh A.B., Cao A., Liu J. (2015). Inhibition of PCSK9 transcription by berberine involves down-regulation of hepatic HNF1alpha protein expression through the ubiquitin-proteasome degradation pathway. J. Biol. Chem..

[49] Widenmaier S.B., Snyder N.A., Nguyen T.B., Arduini A., Lee G.Y., Arruda A.P., Saksi J., Bartelt A., Hotamisligil G.S. (2017). NRF1 Is an ER Membrane Sensor that Is Central to Cholesterol Homeostasis. Cell.

[50] Muller J.A., Gross R., Conzelmann C., Kruger J., Merle U., Steinhart J., Weil T., Koepke L., Bozzo C.P., Read C. (2021). SARS-CoV-2 infects and replicates in cells of the human endocrine and exocrine pancreas. Nat. Metab..

[51] Gkogkou E., Barnasas G., Vougas K., Trougakos I.P. (2020). Expression profiling meta-analysis of ACE2 and TMPRSS2, the putative anti-inflammatory receptor and priming protease of SARS-CoV-2 in human cells, and identification of putative modulators. Redox Biol..

[52] Lyu K., Zhang D., Song J., Li X., Perry R.J., Samuel V.T., Shulman G.I. (2021). Short-term overnutrition induces white adipose tissue insulin resistance through sn-1,2-diacylglycerol/PKCepsilon/insulin receptor Thr1160 phosphorylation. JCI Insight.

[53] Rogers M.A., Liu J., Song B.L., Li B.L., Chang C.C., Chang T.Y. (2015). Acyl-CoA:cholesterol acyltransferases (ACATs/SOATs): Enzymes with multiple sterols as substrates and as activators. J. Steroid Biochem. Mol. Biol..

[54] Miyazaki A., Sakai M., Sakamoto Y., Horiuchi S. (2003). Acyl-coenzyme A:cholesterol acyltransferase inhibitors for controlling hypercholesterolemia and atherosclerosis. Curr. Opin. Investig. Drugs.

[55] Hafiane A., Gianopoulos I., Sorci-Thomas M.G., Daskalopoulou S.S. (2022). Current models of apolipoprotein A-I lipidation by adenosine triphosphate binding cassette transporter A1. Curr. Opin. Lipidol..

[56] Akashi S., Morita A., Mochizuki Y., Shibuya F., Kamei Y., Miura S. (2021). Citrus hassaku Extract Powder Increases Mitochondrial Content and Oxidative Muscle Fibers by Upregulation of PGC-1alpha in Skeletal Muscle. Nutrients.

[57] Li J., Meng Q., Fu Y., Yu X., Ji T., Chao Y., Chen Q., Li Y., Bian H. (2021). Novel insights: Dynamic foam cells derived from the macrophage in atherosclerosis. J. Cell Physiol..

[58] Korber M., Klein I., Daum G. (2017). Steryl ester synthesis, storage and hydrolysis: A contribution to sterol homeostasis. Biochim. Biophys. Acta Mol. Cell Biol. Lipids.

[59] Pal P., Gandhi H., Giridhar R., Yadav M.R. (2013). ACAT inhibitors: The search for novel cholesterol lowering agents. Mini Rev. Med. Chem..

[60] Shibuya K., Kawamine K., Miura T., Ozaki C., Edano T., Mizuno K., Yoshinaka Y., Tsunenari Y. (2018). Design, synthesis and pharmacology of aortic-selective acyl-CoA: Cholesterol O-acyltransferase (ACAT/SOAT) inhibitors. Bioorg. Med. Chem..

[61] Kuroyanagi K., Kang M.S., Goto T., Hirai S., Ohyama K., Kusudo T., Yu R., Yano M., Sasaki T., Takahashi N. (2008). Citrus auraptene acts as an agonist for PPARs and enhances adiponectin production and MCP-1 reduction in 3T3-L1 adipocytes. Biochem. Biophys. Res. Commun..

[62] Abels E.R., Breakefield X.O. (2016). Introduction to Extracellular Vesicles: Biogenesis, RNA Cargo Selection, Content, Release, and Uptake. Cell Mol. Neurobiol..

[63] Jackson C.E., Scruggs B.S., Schaffer J.E., Hanson P.I. (2017). Effects of Inhibiting VPS4 Support a General Role for ESCRTs in Extracellular Vesicle Biogenesis. Biophys. J..

[64] Tavares M.R., Pavan I.C., Amaral C.L., Meneguello L., Luchessi A.D., Simabuco F.M. (2015). The S6K protein family in health and disease. Life Sci..

[65] Muñoz-Ayala A.C.-V.B., García-González V. (2022). Initiation of protein translation, metabolic mechanisms for cancer development, progression and chemoresistance. Advances in Protein Chemistry and Structural Biology.

[66] Krebs J., Agellon L.B., Michalak M. (2015). Ca(2+) homeostasis and endoplasmic reticulum (ER) stress: An integrated view of calcium signaling. Biochem. Biophys. Res. Commun..

[67] Moncan M., Mnich K., Blomme A., Almanza A., Samali A., Gorman A.M. (2021). Regulation of lipid metabolism by the unfolded protein response. J. Cell Mol. Med..

[68] Eizirik D.L., Miani M., Cardozo A.K. (2013). Signalling danger: Endoplasmic reticulum stress and the unfolded protein response in pancreatic islet inflammation. Diabetologia.

[69] Lyu J., Fukunaga K., Imachi H., Sato S., Kobayashi T., Saheki T., Ibata T., Yoshimura T., Iwama H., Murao K. (2021). Oxidized LDL Downregulates ABCA1 Expression via MEK/ERK/LXR Pathway in INS-1 Cells. Nutrients.

[70] Wijesekara N., Kaur A., Westwell-Roper C., Nackiewicz D., Soukhatcheva G., Hayden M.R., Verchere C.B. (2016). ABCA1 deficiency and cellular cholesterol accumulation increases islet amyloidogenesis in mice. Diabetologia.

[71] Bonfleur M.L., Vanzela E.C., Ribeiro R.A., de Gabriel Dorighello G., de Franca Carvalho C.P., Collares-Buzato C.B., Carneiro E.M., Boschero A.C., de Oliveira H.C. (2010). Primary hypercholesterolaemia impairs glucose homeostasis and insulin secretion in low-density lipoprotein receptor knockout mice independently of high-fat diet and obesity. Biochim. Biophys. Acta.

[72] Hao M., Head W.S., Gunawardana S.C., Hasty A.H., Piston D.W. (2007). Direct effect of cholesterol on insulin secretion: A novel mechanism for pancreatic beta-cell dysfunction. Diabetes.

[73] Hsu H., Hsu P., Cheng M.H., Ito Y., Kanda E., Schaefer E.J., Ai M. (2019). Lipoprotein Subfractions and Glucose Homeostasis in Prediabetes and Diabetes in Taiwan. J. Atheroscler. Thromb..

[74] Amemiya-Kudo M., Oka J., Ide T., Matsuzaka T., Sone H., Yoshikawa T., Yahagi N., Ishibashi S., Osuga J., Yamada N. (2005). Sterol regulatory element-binding proteins activate insulin gene promoter directly and indirectly through synergy with BETA2/E47. J. Biol. Chem..

[75] Amemiya-Kudo M., Oka J., Takeuchi Y., Okazaki H., Yamamoto T., Yahagi N., Matsuzaka K., Okazaki S., Osuga J., Yamada N. (2011). Suppression of the pancreatic duodenal homeodomain transcription factor-1 (Pdx-1) promoter by sterol regulatory element-binding protein-1c (SREBP-1c). J. Biol. Chem..

[76] Sun H., Zhang A., Gong Y., Sun W., Yan B., Lei S., Yao L.H. (2022). Improving effect of cordycepin on insulin synthesis and secretion in normal and oxidative-damaged INS-1 cells. Eur. J. Pharmacol..

[77] Dhanya R., Kartha C.C. (2021). Quercetin improves oxidative stress-induced pancreatic beta cell alterations via mTOR-signaling. Mol. Cell Biochem..

[78] Urakami T. (2019). Maturity-onset diabetes of the young (MODY): Current perspectives on diagnosis and treatment. Diabetes Metab. Syndr. Obes..

[79] Yamagata K. (2014). Roles of HNF1alpha and HNF4alpha in pancreatic beta-cells: Lessons from a monogenic form of diabetes (MODY). Vitam. Horm..

