# Supplementary material for: LDL Promotes Disorders in β-Cell Cholesterol Metabolism, Implications on Insulin Cellular Communication Mediated by EVs
Source: Metabolites. 2022 Aug 16;12(8):754. doi: 10.3390/metabo12080754 (PMC9415214; doi:10.3390/metabo12080754)

LDL promotes disorders in β-cells cholesterol metabolism, implications on insulin cellular communication mediated by EVs.

Lizbeth Guevara-Olaya[1,2,#], Brenda Chimal-Vega[1,2,#], César Yahel Castañeda-Sanchez[1,2], Leslie Y. López-Cossio[1,2], Angel Pulido-Capiz[1,3], Octavio Galindo-Hernández[1,2], Raúl Díaz-Molina[1,2], Josefina Ruiz Esparza Cisneros[4], Victor García-González[1,2*].

[1]Departamento de Bioquímica, Facultad de Medicina Mexicali, Universidad Autónoma de Baja California, Mexicali 21000, Baja California, México.
[2]Laboratorio Multidisciplinario de Estudios Metabólicos y Cáncer. Facultad de Medicina Mexicali, Universidad Autónoma de Baja California, Mexicali 21000, Baja California, México.
[3]Laboratorio de Biología Molecular, Facultad de Medicina Mexicali, Universidad Autónoma de Baja California, Mexicali 21000, Baja California, México.
[4]Departamento de Nutrición, Facultad de Medicina, Universidad Autónoma de Baja California, Mexicali 21000, Baja California, México.
[#] Equal contribution.
*To whom correspondence should be addressed.

Supplementary figures

Figure S1. Characterization of fractions LDL isolation was obtained by the KBr-ultracentrifugation method. Western-blot images of apoB and ApoA-1 characterized in plasma, VLDL/IDL, LDL, and HDL fractions, as well as in plasma-free lipoproteins.

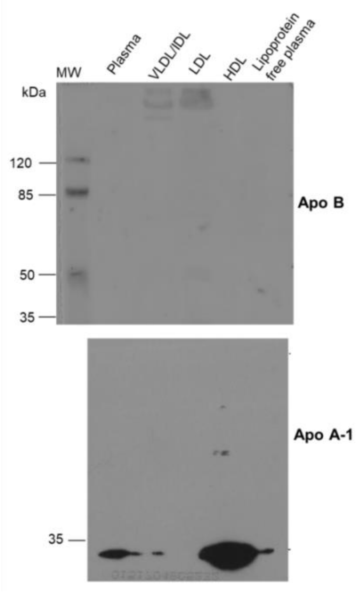

Figure S2. LDL endocytosis in C9 cells (hepatocytes). C9 cells were treated with increasing concentrations of dil-LDL (0-20 μg/mL), representative images showing Hoescht (blue), dil-LDL (red), and merge in each treatment.

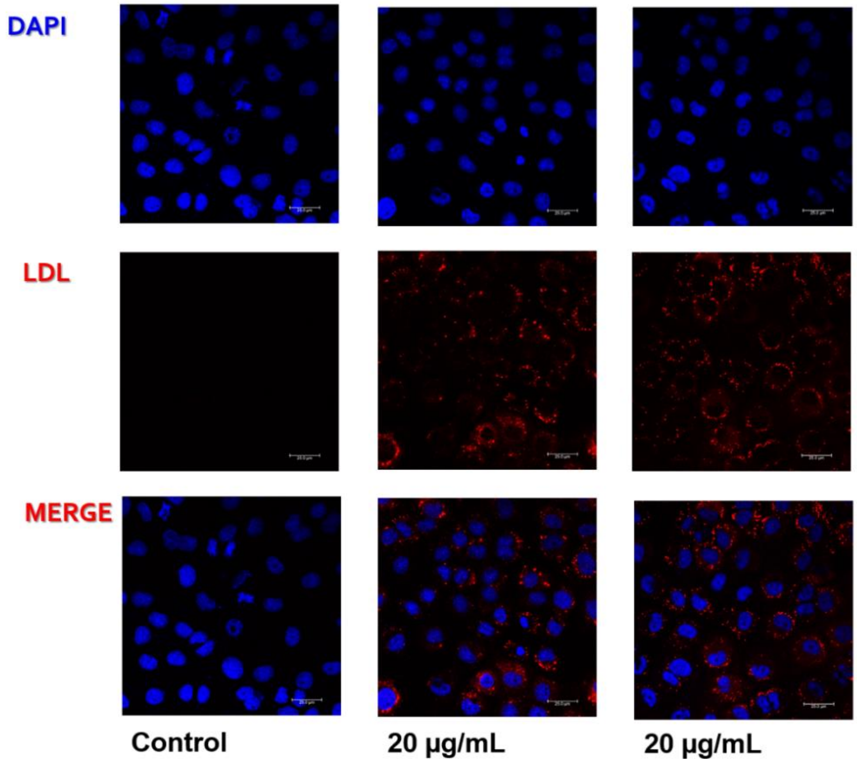

Figure S3. Characterization of endoplasmic reticulum isolates through the evaluation of protein markers PDI, SERCA2 under LDL treatment.

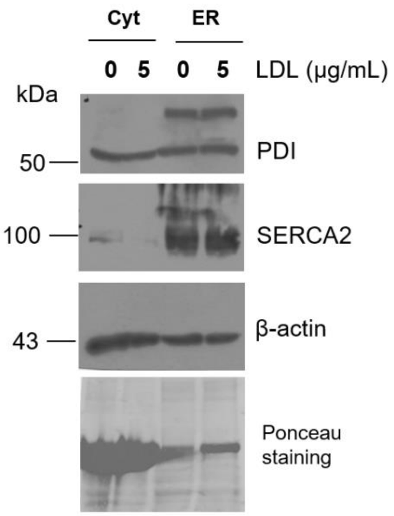

Figure S4. Auraptene (Aur) treatment (4-16 μM) regulates the expression of cholesterol transporter ABCA1 in Langerhans β-cells. Western-blot of ABCA1 in cellular extracts under LDL (20μg/mL) and Aur treatments. Tunicamycin (Tum) was used as an endoplasmic reticulum stress inducer. β-actin was used as a loading control.

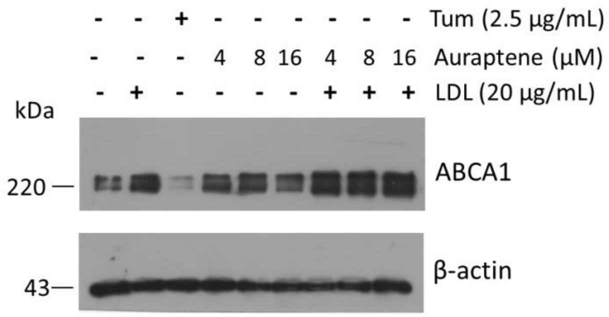

Figure S5 VPS4 expression in Langerhans β-cells under LDL and auraptene (Aur) treatment. Western-blot of VPS4 in cellular extracts under LDL (20μg/mL) and Aur treatments. β-actin was used as a loading control. Quantitative characterization of VPS4, results are expressed as % of control, (n = 3, mean ±SD).

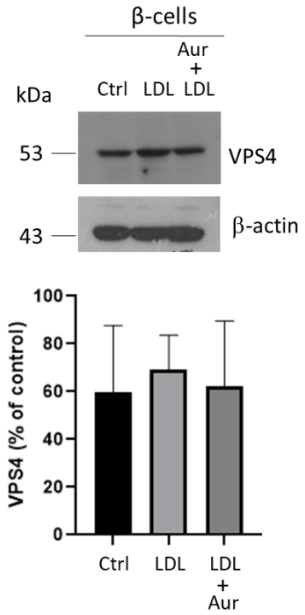

Figure S6. Effect of LDL (20 μg/mL) and insulin (Ins) (28 IU/L) on insulin signaling in hepatocyte cultures through p-p70S6Kα, p70S6Kα, and eIF4E. β-actin was used as a loading control.

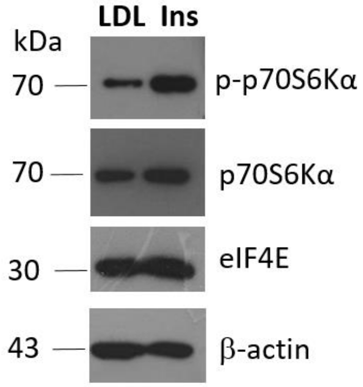

Figure S7. Evaluation of protocols employed in cellular fractionation on Langerhans β-cell cultures. Lamin B1 western-blot detection in samples of protocol A (sucrose/imidazol), protocol B (manitol/sucrose), and protocol C (sucrose gradient/HEPES). Nucleus (N) and cytoplasm (C) are depicted.

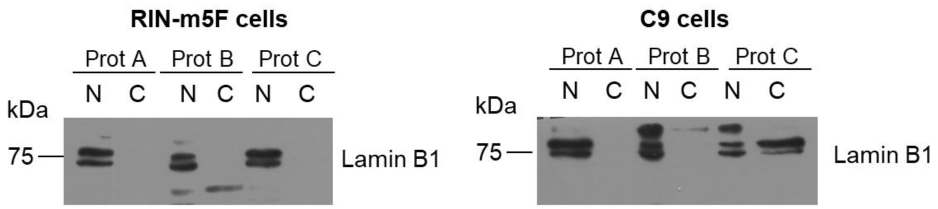

Supplement: Supplementary file 1 [file metabolites-12-00754-s001.zip › metabolites-1845428-supplementary .pdf]
